# Post-interventional Outcomes in the Management of Adult Calcaneonavicular Coalitions: A Systematic Review

**DOI:** 10.7759/cureus.31253

**Published:** 2022-11-08

**Authors:** Hamza Duffaydar, Mohammed Elmajee, Alexander A Dermanis, Shakir Hussain, Anand Pillai

**Affiliations:** 1 College of Medical and Dental Sciences, University of Birmingham, Birmingham, GBR; 2 Trauma and Orthopedics, Royal Wolverhampton NHS Trust, Wolverhampton, GBR; 3 Trauma and Orthopedics, Royal Orthopedic Hospital NHS Foundation Trust, Birmingham, GBR; 4 Trauma and Orthopedics, Worcestershire Acute Hospitals NHS Trust, Birmingham, GBR; 5 Trauma and Orthopedics, University Hospitals Birmingham NHS Foundation Trust, Birmingham, GBR; 6 Trauma and Orthopedics, Wythenshawe Hospital, Manchester University NHS Foundation Trust, Manchester, GBR

**Keywords:** ankle and foot, complications, outcome, tarsal coalition, calcaneonavicular coalition

## Abstract

Calcaneonavicular coalitions in adults can be managed conservatively or through operative means involving resection or arthrodesis of the joints. The aim of this systematic review was to compare complication rates and functional outcomes for the different interventions.

PubMed, MEDLINE, Embase, and the Cochrane Library were searched for relevant studies that reported outcomes for the management of calcaneonavicular coalitions in adults. Twenty-three studies met the inclusion criteria, comprising 118 coalitions. Forty-one coalitions were managed conservatively and 71 through operative means of which, 62 included a resection and nine had an arthrodesis performed. Patients who were operated upon had a significantly higher complication rate of 23.4% compared to 10.6% for those who were managed conservatively (p=0.048). There was no significant difference in complication rates among those who had a resection or an arthrodesis. All studies demonstrated an improvement in functional outcomes regardless of intervention used. Conservative management of calcaneonavicular coalitions in adults should continue to be advocated as first-line treatment given the lower complication rates compared to operative means.

## Introduction and background

Introduction

Tarsal coalition is an abnormal connection between two or more tarsal bones due to a failure of segmentation of the primitive mesenchyme during the development of the foot [1]. The coalition may be fibrous (syndesmosis), cartilaginous (synchondrosis), or osseous (synostosis) and this disorder is inherited through an autosomal dominant pattern [2]. The true incidence of tarsal coalitions is unknown as only about 25% of individuals having tarsal coalition become symptomatic, require investigations, and pursue treatment [2]. A recent cadaveric study has shown an incidence as high as 13% of the population [3]. Although coalitions can occur between any tarsal bones, calcaneonavicular coalitions are the most frequent ones, accounting for 53% of tarsal coalitions [4].

The onset of symptoms in calcaneonavicular coalitions usually occurs between the ages of eight and 12 years when ossification takes place in the pre-existing fibrous coalition [1]. Commonly reported symptoms include anterolateral pain at the ankles, limitations in physical activity, and recurrent sprains to the ankle [5]. The first-line treatment for symptomatic tarsal coalitions is by conservative means. This is usually in the form of activity modification, non-steroidal anti-inflammatory drugs (NSAIDs) for pain relief, orthotics, or support via a walking boot or plaster [6]. However, if conservative measures fail, treatment is through surgical means by either open/arthroscopic resection of the coalition or arthrodesis where there is fusion of the affected joint [7,8]. These procedures are not independent of each other and in cases where resection has failed, patients may have an arthrodesis [7,8].

The literature indicates that surgical resection may be preferred in younger patients with calcaneonavicular coalitions with no evidence of arthritic changes or coalitions in other tarsal joints [9]. However, no literature has compared the effectiveness of the different interventions on an adult population nor looked at the complications patients may experience if they choose one intervention over the other. Adults differ from children in that their feet have achieved skeletal maturity and thus management may differ. Hence, this systematic review aims to compare the complication rates and functional outcomes for the different interventions used in treating calcaneonavicular coalitions in adults as reported in the existing literature.

This article was previously presented as a poster at the Société Internationale de Chirurgie Orthopédique et de Traumatologie (SICOT) Orthopedic World Congress on September 10, 2021.

## Review

Methods

A systematic search of the major databases - PubMed, MEDLINE, Embase, and the Cochrane Library - was conducted by two independent reviewers (HD and AD﻿) in line with the Preferred Reporting Items for Systematic Reviews and Meta-Analyses (PRISMA) guidelines. The search terms used were “tarsal coalition,” “calcaneonavicular coalition” and “outcome,” “results,” and “complications”. The reference lists of included papers were further screened for eligible studies and a manual search of Google Scholar was also conducted. The last search of databases was conducted on February 19, 2021. Only studies with patients aged 16 years or older and published in English were included. Studies that included both patients younger and older than 16 years of age were also included but data was only extracted for those participants 16 years or older. Any disagreement regarding selection of studies was resolved by the general consensus of the two reviewers, and if required, the senior author (ME) was consulted.

Data extracted included patient demographics, number of coalitions, intervention, follow-up time, complications, mean pre-intervention, and post-intervention scores. For studies that did not include a scoring criterion, a symptom score, designed by the authors, was used to assess changes in symptoms and quality of life following intervention (Table 1). Studies were assigned a level of evidence as per the Centre of Evidence-Based Medicine (CEBM) classification [10]. The National Institutes of Health (NIH) “Quality Assessment Tool for case series studies” and the CEBM “Critical Appraisal of a Case Study tool” were used to assess risk of bias in the studies [11,12].

**Table 1:** Symptom score used to assess changes in symptoms and quality of life following intervention. The table is created by the authors of this study.

Score	Extent of symptomatology	Quality of life implication
1	Asymptomatic	Can carry out any activity with no limitation
2	Mild symptomatology	Small degree of activity limitation, i.e., in sports on exertion
3	Moderate symptomatology	Fair degree of activity limitation, i.e., in day-to-day activities on exertion
4	Severe symptomatology	Substantial degree of activity limitation, i.e., in day-to-day activities without exertion
5	Disabling symptomatology	Disabling degree of activity limitation (cannot carry out any activity involving mobilization)

Results

Study Selection

**Figure 1:**
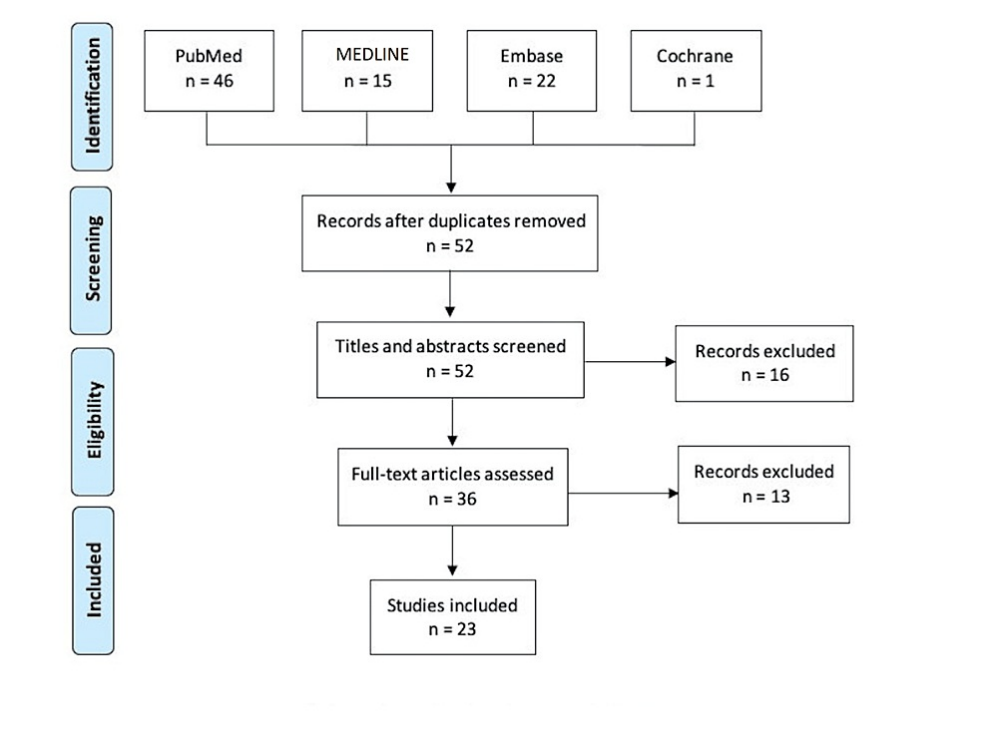
PRISMA flow diagram summarizing study selection. PRISMA: Preferred Reporting Items for Systematic Reviews and Meta-Analyses

The literature search identified 84 studies and after removing duplicates, the titles and abstracts of 52 studies were screened for. The full text of 36 studies was reviewed of which 23 studies met the inclusion criteria [13-35]. Screening of reference lists yielded no additional studies. Figure 1 summarizes the process of study selection.

Characteristics of Included Studies

All included studies were level 4 according to the CEBM classification [10]. Twelve of these studies were case reports [13-24], while 11 were case series [25-35], with a total of 97 patients (118 feet) treated for calcaneonavicular coalition, of these 41 patients were male and 56 were female. The mean age of included patients was 33.6 years with a range from 16 to 81 years. The mean follow-up time of patients post-intervention was 53 months. Table 2 summarizes the demographics of patients included in this study.

**Table 2:** Patient demographics for those included in this systematic review. CN: calcaneonavicular coalition

Study	Design	Level of evidence	Number of patients (number of feet)	Number of coalitions	Sex	Average age in years (range)
Farid and Faber, 2019 [13]	Case report	4	1 (2)	2 CN	1 female	42
Watts et al., 2017 [14]	Case report	4	1 (1)	1 CN	1 male	27
Lalli et al., 2014 [15]	Case report	4	1 (1)	1 CN	1 male	50
Bauer et al., 2010 [16]	Case report	4	1 (1)	1 CN	1 female	27
Acabbo, 2009 [17]	Case report	4	1 (1)	1 CN	1 female	19
Pai et al., 2009 [18]	Case report	4	1 (1)	1 CN	1 male	43
Efstathopoulos et al., 2006 [19]	Case report	4	1 (1)	1 CN	1 male	25
Nilsson and Coetzee, 2006 [20]	Case report	4	1 (1)	1 CN	1 male	47
Plotkin et al., 1996 [21]	Case report	4	1 (2)	2 CN	1 male	17
Tanaka et al., 1995 [22]	Case report	4	1 (1)	1 CN	1 male	23
Richards et al., 1984 [23]	Case report	4	1 (2)	2 CN	1 male	17
Wray and Herndon, 1963 [24]	Case report	4	1 (2)	2 CN	1 male	37
Singh and Parsons, 2012 [25]	Case series	4	2 (2)	2 CN	2 males	26 (24-27)
Scott and Tuten, 2007 [26]	Case series	4	7 (8)	8 CN	2 males, females	41 (31-61)
Varner and Michelson, 2000 [27]	Case series	4	14 (14)	14 CN	N/A	40 (16-81)
Fuson and Barrett, 1998 [28]	Case series	4	25 (28)	28 CN	7 males, 18 females	41(23-56)
Cohen et al., 1996 [29]	Case series	4	12 (13)	13 CN	N/A	33 (19-48)
Gonzalez and Kumar, 1990 [30]	Case series	4	2 (4)	4 CN	N/A	17 (17)
O'Neill and Micheli, 1989 [31]	Case series	4	1 (2)	2 CN	1 male	17
Blockey, 1955 [32]	Case series	4	3 (3)	3 CN	3 males	20 (17-26)
Cain and Hyman, 1978 [33]	Case series	4	3 (3)	3 CN	3 males	20 (17-25)
Rankin and Baker, 1974 [34]	Case series	4	5 (7)	7 CN	5 males	19 (17-22)
Heikel, 1962 [35]	Case series	4	11 (18)	18 CN	9 males, 2 females	27 (16-63)

Intervention

Forty-seven calcaneonavicular coalitions were treated conservatively and this involved modification of activities, physiotherapy exercises, NSAIDs for pain relief, support via a cast, or a combination of the above. Seventy-one calcaneonavicular coalitions were treated operatively of those 62 were resection with or without interposition of soft tissue or bone wax, and nine were triple arthrodesis procedures.

Post-intervention Complications

The overall complication rate was 19.5% (n=23). Tables 3, 4 summarize the complications encountered in each study. The most common complication overall was pain which made up 48% (n=11) of all reported complications. This was followed by wound infection/necrosis and recurrence of the coalition, each making up 13% of reported complications (n=3). Other reported complications included talar fracture (n=2), limited subtalar movement (n=2), gouty arthritis (n=1), and peroneal spasm (n=1).

**Table 3:** Results of case reports on calcaneonavicular coalitions. SS: symptom score, AOFAS: American Orthopedic Foot and Ankle Society, VAS: visual analog scale

Study	Design	Number of coalitions	Approach	Intervention	Mean follow-up in months (range)	Complications	Scoring criteria	Mean pre-intervention score	Mean post-intervention score
Farid and Faber, 2019 [13]	Case report	2 CN	Conservative	Adjustment of activities	12	None	SS	3	1
Watts et al., 2017 [14]	Case report	1 CN	Conservative	Walking boot	4	None	SS	N/A	1
Lalli et al., 2014 [15]	Case report	1 CN	Operative	En-bloc resection	3	None	SS	4	1
Bauer et al., 2010 [16]	Case report	1 CN	Operative	Arthroscopic resection	24	None	AOFAS	23/100	82/100
Acabbo, 2009 [17]	Case report	1 CN	Conservative	Physiotherapy exercises, gait training, balance exercises	2	None	VAS	8/10	0/10
Pai et al., 2009 [18]	Case report	1 CN	Conservative	Plaster	4	None	SS	3	1
Efstathopoulos et al., 2006 [19]	Case report	1 CN	Operative	Triple arthrodesis	32	None	SS	3	1
Nilsson and Coetzee, 2006 [20]	Case report	1 CN	Conservative	Low impact training	6	None	SS	4	1
Plotkin et al., 1996 [21]	Case report	2 CN	Operative	Triple arthrodesis	36	None	SS	4	1
Tanaka et al., 1995 [22]	Case report	1 CN	Operative	En-bloc resection	27	None	SS	4	1
Richards et al., 1984 [23]	Case report	2 CN	Operative	Talonavicular arthrodesis	N/A	Pain	SS	4	2
Wray and Herndon, 1963 [24]	Case report	2 CN	Conservative	Longitudinal arch supports	N/A	N/A	SS	4	1

**Table 4:** Results of case series on calcaneonavicular coalitions. MOXFQ: Manchester Oxford Foot Questionnaire, VAS: visual analog scale, AOFAS: American Orthopedic Foot and Ankle Society, SS: symptom score, ROM: range of movement

Study	Design	Number of coalitions	Approach	Intervention	Mean follow-up in months (range)	Complications	Scoring criteria	Mean pre-intervention score	Mean post-intervention score
Singh and Parsons, 2012 [25]	Case series	2 CN	Operative	Arthroscopic resection	6 (6)	None	MOXFQ	78	42
		-	-	-	-	-	VAS	8	3
Scott and Tuten, 2007 [26]	Case series	8 CN	Operative	En-bloc resection	56.5 (39-84.5)	1 superficial wound infection, 1 dysesthesia	AOFAS	N/A	87 (79-97)
Varner and Michelson, 2000 [27]	Case series	10 CN	Conservative	Cast immobilization, activity modification, and NSAIDs	28 (4 to 62)	1 peroneal spasm	SS	3	2.1
		4 CN	Operative	1 en-bloc resection, 3 arthrodesis		None	SS	3	1
Fuson and Barrett, 1998 [28]	Case series	2 CN	Conservative	Activity modification, NSAIDs, and orthotics	120 (120)	None	Good, fair, or poor	N/A	2 Good
		26 CN	Operative	En-bloc resection		1 gouty arthritis, 3 reflex sympathetic dystrophy		N/A	21 good, 1 fair, 4 poor
Cohen et al., 1996 [29]	Case series	13 CN	Operative	En-bloc resection	36 (17-54)	1 reflex sympathetic dystrophy, 2 marginal wound necrosis, 2 required further arthrodesis	ROM/degrees	3.33	14.17
Gonzalez and Kumar, 1990 [30]	Case series	4 CN	Operative	En-bloc resection	78 (36-120)	2 talar breaking, 1 pain, 1 limited subtalar movement	SS	4.5	3.5
O'Neill and Micheli, 1989 [31]	Case series	2 CN	Operative	En-bloc resection	95 (95)	1 recurrence of bar	AOFAS	54	74
Blockey, 1955 [32]	Case series	3 CN	Conservative	Plaster and physiotherapy	24 (24)	1 limited inversion, 2 rigidity and pain	SS	4	2
Cain and Hyman, 1978 [33]	Case series	3 CN	Operative	En-bloc resection	60 (60-60)	1 pain	SS	4	1
Rankin and Baker, 1974 [34]	Case series	7 CN	Conservative	Rest or activity modification	8 (8)	None	SS	4	1
Heikel, 1962 [35]	Case series	17 CN	Conservative	Arch support and rest	12 (6-18)	1 pain	Good, fair, or poor	N/A	9 good, 1 poor
		1 CN	Operative	Arthrodesis	6 (6)	None	-	N/A	1 good

Conservative vs Operative Complications

The post-intervention complication rate in coalitions managed conservatively was significantly lower (10.6%) compared to those managed operatively (23.4%) and the difference was statistically significant (χ2=3.90, p=0.048). However, in both groups, pain remains the most commonly reported complication, reported in 6% (n=3) of interventions managed conservatively and 11% (n=8) of operative procedures. Other complications reported were peroneal spasm (n=1) and limited foot inversion (n=1) in interventions managed conservatively and wound infection (n=3), further arthrodesis required (n=3), talar fracture (n=2), gouty arthritis (n=1), and limited foot inversion (n=1) in operative procedures.

Resection vs Arthrodesis Complications

The complication rate of resection and arthrodesis were 27.4% and 11.1%, respectively. However, the difference in complication rates was not statistically significant (χ2=1.10, p=0.293). The only reported complication in arthrodesis was pain (n=1) but other complications reported in resection included wound infection (n=3), requiring operative procedure (n=3), talar fracture (n=2), gouty arthritis (n=1), and limited foot inversion (n=1). It is worth noting that in studies where an arthroscopic resection was performed (n=3), there were no reported complications. However, in studies where open en-bloc resection was performed (n=59), complication rate was 28.8%. Again, the difference in complication rates between the two resection procedures was not statistically significant (χ2=0.85, p=0.355).

Functional Outcomes

A total of six scoring criteria were used to report the outcomes including the symptom score designed by the authors. Irrespective of the scoring criteria used or intervention, all studies reported an improvement in functional outcomes following intervention. The pre- and post-intervention outcome scores are also summarized in Tables 3, 4.

Conservative vs Operative Outcomes

There was a mean improvement in the symptom score by 1.85/5 in 27 studies that used a conservative intervention as compared to a mean improvement of 2.16/5 in 18 studies that used operative means of intervention. Where the VAS scoring criteria were used, the study by Acabbo which used a conservative approach reported an improvement by 8/10 whereas two studies where patients had operative interventions reported a mean improvement of 2/10 [17]. In studies that reported outcomes as good, fair, or poor, there were 11 good and one poor where conservative approaches were used (n=12) and 22 good, one fair, and four poor where operative approaches were used (n=27).

Resection vs Arthrodesis Outcomes

The mean improvement in symptom score for studies with resection was 2.1/5 (n=10), as compared to 2.3/5 for studies where a triple arthrodesis was performed (n=8). There were 21 good, one fair, and four poor outcomes reported in studies with resection and one good outcome reported in the study by Heikel where a triple arthrodesis was performed and such a scoring criterion was used [35]. Other scoring criteria used in studies with resection were the AOFAS with a mean improvement of 40.5/100, the MOXFQ with a mean improvement of 36/100, and the VAS criteria with a mean improvement of 5/10. Cohen et al. also reported an average improvement in range of ankle movement by 10.8° in 13 patients where resection was performed [29].

Risk of Bias

A summary of risk of bias assessment is outlined in Table 5 and Table 6 for case reports and case series, respectively. Two case reports had a quality rating of “good,” seven “fair,” and three “poor” as per the CEBM guidelines [12]. Three case series had a quality rating of “good,” six “fair,” and two “poor” as per the NIH quality rating tool [11].

**Table 5:** Critical appraisal of case reports.

Study	Did the study address a clearly focused question /issue?	Is the research method (study design appropriate for answering the research question?	Are both the setting and the subjects representative with regard to the population to which the findings will be referred?	Is the researcher’s perspective clearly described and taken into account?	Are the methods for collecting data clearly described?	Are the methods for analyzing the data likely to be valid and reliable? Are quality control measures used?	Was the analysis repeated by more than one researcher to ensure reliability?	Are the results credible, and if so, are they relevant for practice?	Are the conclusions drawn justified by the results?	Are the findings of the study transferable to other settings?	Overall quality rating
Farid and Faber, 2019 [13]	Yes	Yes	Yes	Yes	N/A	Yes	N/A	Yes	Yes	Yes	Fair
Watts et al., 2017 [14]	Yes	Yes	Yes	Yes	N/A	Yes	N/A	Yes	Yes	Yes	Fair
Lalli et al., 2014 [15]	Yes	Yes	Yes	Yes	N/A	Yes	N/A	Yes	Yes	Yes	Fair
Bauer et al., 2010 [16]	Yes	Yes	Yes	Yes	Yes	Yes	N/A	Yes	Yes	Yes	Good
Acabbo, 2009 [17]	Yes	Yes	Yes	Yes	Yes	Yes	N/A	Yes	Yes	Yes	Good
Pai et al., 2009 [18]	Yes	Yes	Yes	Yes	N/A	Yes	N/A	Yes	Yes	Yes	Fair
Efstathopoulos et al., 2006 [19]	Yes	Yes	Yes	Yes	N/A	Yes	N/A	Yes	Yes	Yes	Fair
Nilsson and Coetzee, 2006 [20]	Yes	Yes	Yes	Yes	N/A	Yes	N/A	Yes	Yes	Yes	Fair
Plotkin et al., 1996 [21]	Yes	Yes	Yes	Yes	N/A	Yes	N/A	Yes	No	Yes	Poor
Tanaka et al., 1995 [22]	Yes	Yes	Yes	Yes	N/A	Yes	N/A	Yes	Yes	Yes	Fair
Richards et al., 1984 [23]	Yes	Yes	Yes	Yes	N/A	Yes	N/A	Yes	No	Yes	Poor
Wray and Herndon, 1963 [24]	Yes	Yes	Yes	Yes	N/A	Yes	N/A	Yes	No	Yes	Poor

**Table 6:** Critical appraisal of case series.

Study	Was the study question or objective clearly stated?	Was the study population clearly and fully described, including a case definition?	Were the cases consecutive?	Were the subjects comparable?	Was the intervention clearly described?	Were the outcome measures clearly defined, valid, reliable, and implemented consistently across all study participants?	Was the length of follow-up adequate?	Were the statistical methods well-described?	Were the results well-described?	Overall quality rating
Singh and Parsons, 2012 [25]	Yes	Yes	No	No	Yes	Yes	No	Yes	Yes	Poor
Scott and Tuten, 2007 [26]	Yes	Yes	Yes	Yes	Yes	Yes	Yes	Yes	Yes	Good
Varner and Michelson, 2000 [27]	Yes	Yes	No	Yes	Yes	Yes	Yes	No	Yes	Fair
Fuson and Barrett, 1998 [28]	Yes	Yes	Yes	Yes	Yes	Yes	N/a	No	Yes	Fair
Cohen et al., 1996 [29]	Yes	Yes	No	Yes	Yes	Yes	Yes	Yes	Yes	Good
Gonzalez and Kumar, 1990 [30]	Yes	Yes	No	Yes	Yes	Yes	Yes	No	Yes	Fair
O'Neill and Micheli, 1989 [31]	Yes	Yes	No	Yes	Yes	Yes	Yes	Yes	Yes	Good
Blockey, 1955 [32]	Yes	Yes	No	No	Yes	Yes	Yes	No	Yes	Fair
Cain and Hyman, 1978 [33]	Yes	Yes	No	Yes	Yes	Yes	Yes	No	Yes	Fair
Rankin and Baker, 1974 [34]	Yes	Yes	No	Yes	Yes	Yes	No	No	Yes	Poor
Heikel, 1962 [35]	Yes	Yes	No	No	Yes	Yes	Yes	No	Yes	Fair

Discussions

This study is the first systematic review of the literature to assess complication rates and changes in patient-reported outcome scores following intervention in the management of calcaneonavicular coalitions in the adult population. The overall complication rate following intervention was 19.5% and all studies reported an improvement in outcome scores following intervention regardless of whether a conservative or operative approach was used.

The most commonly reported complication post-intervention was pain, reported in 48% of cases. The results of our study show that patients who were subject to an operative procedure were significantly more likely to encounter complications post-intervention as opposed to those where a conservative means of treatment was used. This, therefore, supports the general consensus that calcaneonavicular coalitions should be managed conservatively and operative procedures attempted only if the former fails [6-8]. There was no identifiable difference in complication rates between resection and arthrodesis nor between open and arthroscopic resection. However, marginal wound necrosis, a major complication, was reported by Cohen et al. only when extensor digitorum brevis (EDB) interposition was attempted following open en-bloc resection of the coalition to prevent reoccurrence. Cohen et al. believe that this may have been caused by an increased area of dead space created by transfer of the muscle leading to necrosis. This complication was not reported again once the technique of EDB interposition was discontinued on the remaining patients operated upon in their study [29]. Patients who had an arthroscopic resection, a relatively new technique first attempted by Lui et al. in 2006, reported no complications as opposed to those who had an open resection [36]. The potential advantages of this technique over open surgery include a swifter post-operative recovery, reduced post-operative pain, more aesthetically appealing for the patient, and lower rates of infection [37].

The first line treatment for calcaneonavicular coalitions is conservative management followed by operative means if there is no improvement [6]. It is generally agreed that resection of the coalition is more suitable unless there is evidence of further coalitions or arthritic changes in the joints [9]. In those cases, or where resection has failed, arthrodesis is recommended [7,8]. In all studies included in this systematic review, the recommended order of treatment was followed except in the study by Tanaka et al. where conservative treatment was not attempted because the patient wanted an early recovery [22]. The improvement in functional outcome scores for all patients in our review was similar between conservative vs operative and resection vs arthrodesis. However, it was difficult to undertake further analysis given the difference in scoring systems used throughout.

Following early reports of recurrence rates as high as 30% in patients having open en-bloc resection of tarsal coalitions, the concept of tissue interposition at the site of resection became popular [38]. Mubarak et al. reported a reduction in calcaneonavicular recurrence to 13% when a fat graft was interposed at site of resection [9]. Similarly, Masquijo et al. reported a recurrence rate of 4% with fat graft, 6% with bone wax, and 40% with EDB interposition [39]. However, both these studies were conducted on a pediatric population, and to date, there is no agreed consensus on the effectiveness of using grafts in an adult population. Hence, our review included a mixture of both patients who had a graft interposition and those that did not. In a systematic review comparing arthroscopic resection of tarsal coalitions, Malik-Tabassum et al. suggest that arthroscopic procedures may also reduce rates of recurrence due to earlier mobilization and weight-bearing post-operatively, inhibiting re-ossification at the site of resection [5]. This however remains to be proven by future studies.

There are several limitations to our study. Most notably, the review only consisted of studies with level 4 tier of evidence and did not identify any randomized control or comparative studies. However, this is due to the paucity of literature surrounding the topic. Furthermore, given the heterogeneity in scoring criteria used in included studies, further statistical analysis on improvement in functional outcome post-intervention was not possible. Lastly, human factors such as the surgeon’s experience in undertaking tarsal resection or arthrodesis should be taken into account when considering post-operative complications or outcomes. This would be difficult to quantify but may be worth taken into consideration.

The strengths of this study are its use of a systematic methodology with a comprehensive database search and vigorous quality assessment of included studies. To date, it remains the first systematic review to assess complications and functional outcomes in the management of calcaneonavicular coalitions within an adult population.

This study has identified the need for further research in the management of adults presenting with calcaneonavicular coalitions. Given that such presentation is relatively less common than in children, it is not surprising that majority of the existing literature are on a pediatric population. However, management and complication rates may differ, hence the need for prospective trials with comparable patient-reported functional outcome scores and follow-up time.

## Conclusions

Conservative management of calcaneonavicular coalitions in adults should continue to be advocated as first-line treatment due to a significantly lower post-intervention complication rate compared to operative procedures. Complication rates following surgery were similar in resection and arthrodesis. Both conservative and operative interventions reported similar improvement in patient-reported functional outcome scores. Future studies on the management of calcaneonavicular coalitions in adults are warranted.
